# Impact of additional HEPA filter on APAP performance and CPAP pressure level in simulated sleep apnea events

**DOI:** 10.3389/fmedt.2022.891390

**Published:** 2022-07-27

**Authors:** Nils Correvon, Lucas Fasquel, Pouyan Yazdani, Jean-Bernard Michotte, Jonathan Dugernier, Olivier Contal

**Affiliations:** ^1^School of Health Sciences (HESAV), HES-SO University of Applied Sciences and Arts Western Switzerland, Lausanne, Switzerland; ^2^Physiotherapy Unit, Réseau Hospitalier Neuchâtelois (RHNe), Pourtalès Hospital, Neuchâtel, Switzerland; ^3^Physiotherapy Unit, Clinique Romande de Réadaptation (CRR), Sion, Switzerland

**Keywords:** CPAP, APAP, obstructive sleep apnea syndrome (OSAS), HEPA filter, antibacterial filter

## Abstract

**Background:**

CPAP is the first line treatment of obstructive sleep apnea. Recently, the use of added filters has been debated following the field safety notice of Philips Respironics™ on potential health risks due to foam degradation used in their ventilators. However, the added resistance of filters has never been analyzed.

**Objectives:**

The primary aim was to investigate the impact of four different filters on APAP mode performance with and without added unintentional air leaks (UIAL) with two simulated respiratory events. The secondary aim was to assess the pressure drop due to the increased filter resistance at different fixed CPAP pressure levels.

**Method:**

This is a bench study. Performance tests were performed on a breathing simulator (ASL 5000™) with a DreamStation™ device. To assess the combined effect of UIAL, a controlled valve was added to the setup.

**Results:**

Without UIAL, the algorithm was able to detect respiratory events and increase pressure level consequently. In the presence of UIAL, the device's response to simulated events was affected. In fixed CPAP mode, the median measured end-expiratory pressure was 6.2 to 10.0% (*p* < 0.001) below the set pressure with the additional filters. Additional UIAL severely impacted the delivered pressure with a median reduction up to 28.3% (*p* < 0.001) to the set pressure.

**Conclusion:**

Despite a slight pressure drop, the APAP algorithm still performed with additional filters when UIAL were avoided. However, the combined effect of added filter resistance and UIAL severely impacted APAP performance and effectively delivered set pressure.

## Introduction

Continuous positive airway pressure (CPAP) is the first line treatment for obstructive sleep apnea (OSA) (1). The automatic positive airway pressure (APAP) mode has also been increasingly used in the last two decades to facilitate pressure level titration (2, 3). Recently, the use of added high-efficiency particulate air (HEPA) filters has been recommended in certain situations to protect users from several causes, but its use and potential effects are still debated (4–6).

Following the recall notification for some Philips Respironics CPAP devices, the FDA and some professional associations firstly mentioned the possibility to use an additional HEPA filter on the respiratory circuit to block larger solid particles as an alternative to discontinue the therapy if the device could not immediately be replaced (4, 6). However, in their update declaration of the 12th of November 2021, the FDA changed their statement and recommended not to use an additional filter with CPAP machine as a HEPA filter could not block some of the harmful chemicals which are off-gassed in the degradation process of the sound abatement foam. The FDA also warns about the increased filter resistance which might impact device's performance (5). The Swiss Pulmonology Society also warns against the same potential issues in case of the use of additional filters and mentions filters could potentially be used with strict caution (6). The alteration of performance might be particularly true for APAP mode as the pressure level adaptation relies on the algorithm's abilities to detect respiratory events.

The recommendation of adding an inline HEPA filter however is still valid in the context of the COVID-19 pandemic and has been made to prevent bacteria and viruses from entering the CPAP tubes and masks, thus protecting the user (7). Although it is known that APAP mode performance is affected by unintentional leaks (3, 8), the added resistance of HEPA filters has never been analyzed on the ability of APAP mode to perform as usual.

This bench study aims to investigate the impact of four different antibacterial filters on APAP mode performance with and without added unintentional air leaks (UIAL) with two simulated respiratory events (i.e., obstructive apnea and hypopnea events). The secondary aim was to assess the pressure drop due to the increased filter resistance at different fixed CPAP pressure levels.

In APAP mode without UIAL, our hypothesis was to observe a slight pressure drop due to the increased filter resistance but a preserved ability to detect respiratory events and adjust pressure. With the presence of UIAL, we expected an altered ability to detect respiratory events and therefore to adjust pressure.

According to the ISO 80601-2-70:2020 standard, the accuracy of the airway pressure measurement shall not be worse than 6% for a max set pressure at 20 cmH_2_O (9). In fixed CPAP mode, we expected to find the pressure drop due to the filter resistance within this range without UIAL and out of this range with UIAL.

## Method

### Bench test configuration

Performance of the DreamStation™ (Philips Respironics™, Murrysville USA) APAP mode and pressure loss from increased filter resistance with fixed CPAP mode were analyzed on a bench test with reproducible and standardized conditions. The Active Servo Lung (ASL) 5000™ simulator (IngMar Medical™, Pittsburgh, USA) was used in this study. A Starling resistor simulating upper airways was added to the system to simulate obstructive apnea and hypopnea events by reducing or abolishing airflow with the use of a syringe to increase pressure into a hermetic tube until the artificial airway placed within collapses partially or totally depending on the type of simulated events. Its combined use with the ASL 5000™ has been already used in a previous study to simulate apnea and hypopnea events (10).

The ASL 5000™ is an active artificial lung that responds to set characteristics. The dedicated software (IngMar Medical™ ASL 5000™ 3.6 version) was used to read and analyze the recorded scenario.

Four different filters were tested in this study. They are listed below, with the resistance announced by the manufacturer:

- GVS™ Medguard™ (GVS Filter Technology™, Morecambe, UK), resistance at 30 L/min (or 0.5 L/s): 0.63 cmH_2_O
- Vyaire™ AirLife™ (Vyaire Medical Inc™., Mettawa, USA), Resistance at 60 L/min: 0.54 cmH_2_O (resistance at 30 L/min is not mentioned)
- GVS™ ECO filter™ 4,222/701 (for ResMed™) (GVS Filter Technology™, Morecambe, UK), resistance at 30 L/min: 1 cmH_2_O
- King System™ Virobac™ II (King Systems Corporation™, Noblesville, USA), resistance at 30 L/min: 0.7 cmH_2_O.

To assess the combined effect of increased filter resistance and UIAL, a UIAL valve was added to the setup. The UIAL valve was developed by Haute Ecole d'Ingénierie de Genève (HEPIA). The device is connected to a computer and a software (Microsoft™ Visual Basic 6.0; Microsoft™ Corporation) which controls the opening diameter of the valve between 0 and 10 mm. Maximal achievable air leaks flow is 60 L/min for a 25 cmH_2_O pressure. In this study, it was decided to set the maximal diameter, at 10 mm.

**Figure 1:**
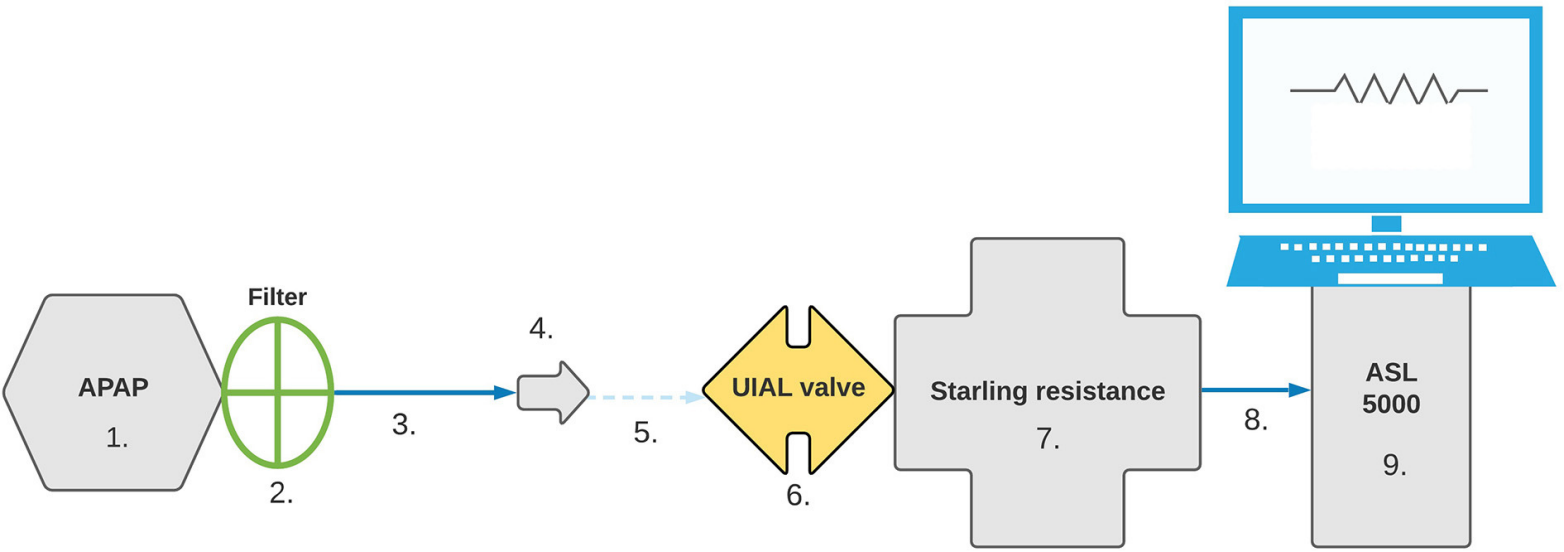
Bench test setup. Configuration was set in the following order: 1. APAP device, 2. Filter, 3. 180 cm SlimLine™ tubing (ResMed™, San Diego, USA), 4. Whisper Swivel™ II exhalation port (Philips-Respironics™, Murrysville, USA), 5. Connector, 6. UIAL valve, 7. Starling Resistance, 8. Connector, 9. ASL 5000™ simulator.

To ensure standardized and reproducible data, a similar configuration was used for simulated obstructive apnea, hypopnea, and fixed CPAP pressure scenarios. Detailed setup can be found in Figure 1.

### Protocol

To assess APAP performance, the mode was set with the following setting: pressure range from 4 to 20 cmH_2_O, no ramp, and Mask type: facial mask. To assess the pressure drop due to increased filter resistance, end-expiratory pressure was measured for every cmH_2_O from 4 to 20 cmH_2_O with fixed CPAP mode.

Respiratory mechanics were set as followed on the ASL 5000™: Compliance: 80 mL/cmH_2_O, Resistance: 5 cmH_2_O/L/s, Inspiratory pressure: 7 cmH_2_O, Frequency: 12 breaths per minute and Inspiratory time 30% of breathing cycle. The tidal volume obtained with these parameters was 500 mL.

For APAP mode analysis, every record started with 2 min of steady breathing (i.e., no events). Then a 25 s event occurred every minute for the rest of the scenario. Therefore, 11 events appeared in each 13 min scenario. Each scenario was repeated twice, without UIAL and with UIAL, to ensure consistent device response. End expiratory pressure data were recorded during the second scenario.

For hypopnea, the syringe of Starling resistance was used to increase pressure into the hermetic tube and reduce airflow. The injected air volume was adapted to ensure a ≥50% airflow diminution as measured by the ASL 5000™ simulator. According to the APAP test, it varied from 2 to 4.5 mL. For obstructive apnea, 20 mL were injected through the syringe to collapse the simulated upper airways and airflow cessation was checked from the ASL 5000™ reading.

### Analysis

Pressure variations were obtained and analyzed with the ASL 5000™ software (version 3.6). End expiratory pressure data were reported once every minute from the second minute to the thirteenth (i.e., every 60 s, after each event, from the 120 s to the 780 s) for each APAP scenario. End-expiratory pressure data were reported at each set pressure level in CPAP mode once the end-expiratory pressure stabilized (e.g., usually after two or three simulated breaths).

Descriptive statistics are expressed as median values and interquartile range. Inferential statistics were performed using a paired samples Wilcoxon test to compare effective measured pressure to the device set pressure in fixed CPAP mode.

## Results

A total of 20 APAP scenarios were recorded (i.e., simulated apnea and hypopnea, without and with each filter type, and with and without UIAL) and 170 end expiratory pressure levels at fixed CPAP were measured (from 4 to 20 cmH_2_O, without and with each filter, with and without UIAL). UIAL were measured at the minimum and maximum set pressure level and ranged from 24 L/min at 4 cmH_2_O pressure to 56.4 L/min at 20 cmH_2_O. Detailed UIAL at each pressure level are found in Table 1.

**Table 1:** Air leaks flow of the UIAL valve according to device set pressure level.

**Pressure (cmH**_2_ **O)**	**4**	**5**	**6**	**7**	**8**	**9**	**10**	**11**	**12**	**13**	**14**	**15**	**16**	**17**	**18**	**19**	**20**
Air leaks flow (L/min)	24.0	27.6	30.0	32.4	34.8	37.2	39.6	42.0	43.2	45.6	48.0	49.2	50.4	51.6	54.0	55.2	56.4

**Figure 2:**
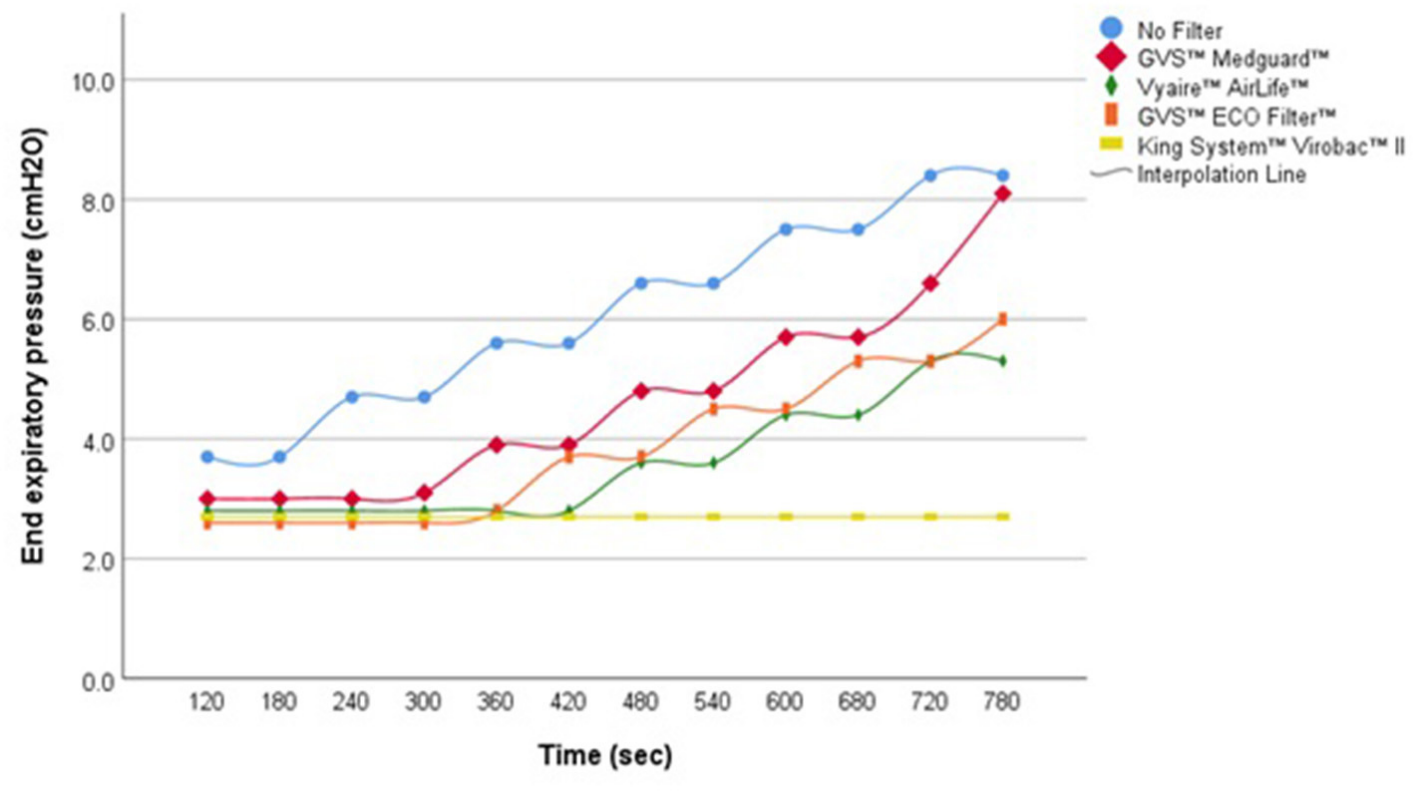
Pressure variation in APAP mode during simulated apnea events, with UIAL.

During simulated apnea and hypopnea events without UIAL, the APAP algorithm was able to detect the respiratory events and to increase pressure level consequently, with a pressure adjustment approximately every two simulated events. In presence of UIAL, the ability of the algorithm to detect simulated respiratory events and to adjust pressure level consequently was variable. An increased delay of pressure adjustment was observed with every filter but one. GVS™ Medguard™ filter performed the best when King System™ Virobac™ II did not enable the algorithm to detect any event during simulated apnea scenarios (Figure 2). Even without filters, Philips Respironics™ APAP algorithm performance was altered, and pressure adjustment was delayed during the simulated hypopnea events with the presence of UIAL. Detailed results are found in Table 2.

**Table 2:** Pressure variation in APAP mode during simulated respiratory events.

**Effective end expiratory pressure (cmH_2_O)**	**Time (s)**			**120**	**180**	**240**	**300**	**360**	**420**	**480**	**540**	**600**	**660**	**720**	**780**
	Without UIAL	Obstructive sleep apnea	No filter	4.0	4.0	4.9	4.9	6.0	6.0	7.0	7.0	7.9	7.9	8.9	8.9
			Filter 1	3.7	3.7	4.6	4.6	5.6	5.6	6.6	6.6	7.5	7.5	8.5	8.5
			Filter 2	3.6	3.6	3.6	4.5	4.5	5.4	5.4	6.4	6.4	7.3	7.3	8.3
			Filter 3	3.5	3.5	3.5	4.4	4.4	5.3	5.3	6.2	6.2	7.2	7.2	8.1
			Filter 4	3.5	3.5	3.5	4.4	4.4	5.4	5.4	6.3	6.3	7.3	7.3	8.2
		Hypopnea	No filter	4.0	4.0	4.9	4.9	6.0	6.0	7.0	7.0	7.9	7.9	8.9	8.9
			Filter 1	3.6	3.6	3.6	4.6	4.6	5.6	5.6	6.5	6.5	7.5	7.5	8.5
			Filter 2	3.6	3.6	4.6	4.6	5.5	5.5	6.5	6.5	7.5	7.5	8.5	8.5
			Filter 3	3.5	3.5	4.4	4.4	5.3	5.3	6.2	6.2	7.1	7.1	8.1	8.1
			Filter 4	3.5	4.4	4.4	5.3	5.3	6.3	6.3	7.2	7.2	8.1	8.1	9.1
	With UIAL	Obstructive sleep apnea	No filter	3.7	3.7	4.7	4.7	5.6	5.6	6.6	6.6	7.5	7.5	8.4	8.4
			Filter 1	3.0	3.0	3.0	3.1	3.9	3.9	4.8	4.8	5.7	5.7	6.6	8.1
			Filter 2	2.8	2.8	2.8	2.8	2.8	2.8	3.6	3.6	4.4	4.4	5.3	5.3
			Filter 3	2.6	2.6	2.6	2.6	2.8	3.7	3.7	4.5	4.5	5.3	5.3	6.0
			Filter 4	2.7	2.7	2.7	2.7	2.7	2.7	2.7	2.7	2.7	2.7	2.7	2.7
		Hypopnea	No filter	3.7	3.7	3.7	3.7	4.7	4.7	5.7	5.7	6.6	6.6	7.5	7.5
			Filter 1	3.1	3.1	3.9	3.9	4.8	4.8	4.8	5.7	5.7	6.5	6.5	7.4
			Filter 2	2.8	2.8	3.6	3.6	4.4	4.4	5.2	5.2	6.1	6.1	6.9	6.9
			Filter 3	2.5	2.5	3.3	3.3	4.0	4.0	4.8	4.8	4.8	5.5	5.5	6.3
			Filter 4	2.7	2.7	3.5	3.5	4.3	4.3	5.1	5.1	5.9	5.9	6.7	6.7

*UIAL, unintentional air leaks; Filter 1, GVS™ Medguard; Filter 2, Vyaire™ AirLife™; Filter 3, GVS™ ECO filter™; Filter 4, King System™ Virobac II™*.

In fixed CPAP mode, the median measured end-expiratory pressure was 1.5 (IQR 0.4)% (*p* < 0.001) below the set pressure without filter and without UIAL within a range of 4 to 20 cmH_2_O set pressure. With the different filters, the average end-expiratory pressure ranged from −6.2 (IQR 2.0) to −10.0 (IQR 1.9)% (*p* < 0.001) to the set pressure. UIAL strongly impacted the delivered pressure. GVS™ Medguard™ filter performed the best with median end-expiratory pressure 16.9 (IQR 2.3)% (*p* < 0.001) below the set pressure. GVS™ ECO Filter™ performed the worst with a median pressure drop of −28.3 (IQR 3.3)% (*p* < 0.001). Detailed results are found in Table 3.

**Table 3:** Effective end-expiratory pressure at different set CPAP pressure level.

**CPAP set pressure (cmH**_2_ **O)**		**4.0**	**5.0**	**6.0**	**7.0**	**8.0**	**9.0**	**10.0**	**11.0**	**12.0**	**13.0**	**14.0**	**15.0**	**16.0**	**17.0**	**18.0**	**19.0**	**20.0**	**Median diff. (IQR), %**
Effective end expiratory pressure (cmH_**2**_O), without UIAL	No filter	4.0	4.9	5.9	6.9	7.9	8.8	9.8	10.8	11.8	12.8	13.8	14.8	15.8	16.8	17.7	18.8	19.7	−1.5 (0.4)*
	Filter 1	3.6	4.6	5.5	6.4	7.4	8.3	9.3	10.3	11.3	12.2	13.2	14.1	15.1	16.0	17.0	18.0	18.9	−6.2 (2.0)*
	Filter 2	3.5	4.5	5.4	6.3	7.2	8.2	9.1	10.1	11.0	12.0	12.9	13.9	14.9	15.8	16.7	17.6	18.6	−8.2 (2.4)*
	Filter 3	3.5	4.4	5.3	6.1	7.1	8.0	9.0	9.8	10.8	11.7	12.7	13.6	14.5	15.5	16.4	17.3	18.3	−10.0 (1.9)*
	Filter 4	3.5	4.4	5.3	6.2	7.1	8.1	9.0	10.0	10.9	11.8	12.8	13.7	14.7	15.6	16.6	17.6	18.4	−9.2 (2.6)*
Effective end expiratory pressure (cmH_**2**_O), with UIAL	No filter	3.8	4.7	5.7	6.6	7.6	8.5	9.4	10.4	11.3	12.3	13.1	14.1	15.0	16.0	16.9	17.8	18.8	−5.9 (0.5)*
	Filter 1	3.1	4.0	4.8	5.6	6.5	7.4	8.2	9.1	10.0	10.8	11.7	12.5	13.4	14.4	15.2	16.0	16.9	−16.9 (2.3)*
	Filter 2	2.9	3.7	4.5	5.3	6.1	7.0	7.8	8.7	9.4	10.3	11.1	12.0	12.8	13.7	14.5	15.3	16.1	−20.9 (3.4)*
	Filter 3	2.6	3.3	4.0	4.8	5.5	6.3	7.1	7.8	8.6	9.4	10.1	10.8	11.6	12.3	13.1	13.8	14.6	−28.3 (3.3)*
	Filter 4	2.7	3.5	4.3	5.1	6.0	6.7	7.6	8.4	9.3	10.0	10.9	11.7	12.5	13.3	14.1	15.0	15.9	−23.1 (3.5)*

CPAP, Continuous positive airway pressure; UIAL, unintentional air leaks; Median diff., the median difference between effective and set pressure at all pressure level; IQR, interquartile range; Filter 1, GVS™ Medguard™; Filter 2, Vyaire™ AirLife™; Filter 3, GVS™ ECO filter™; Filter 4, King System™ Virobac II™, *p-value < 0.001.

## Discussion

In APAP mode, the results showed that, despite a slight pressure difference, the algorithm was able to detect respiratory events and increase pressure level accordingly when respiratory events were simulated. The pressure difference at the end of each scenario was mainly due to the pressure drop caused by the increased filter resistance. However, the combined effects of added resistance and UIAL demonstrated that the type of filter strongly influenced the algorithm's ability to respond to simulated events. The APAP algorithm was not able to detect simulated apnea events with the King System™ Virobac™ II filter and added UIAL. In this situation, the algorithm might not be able to differentiate obstructive events from central events and therefore not adapt pressure level accordingly. Overall, Philips Respironics™ APAP algorithm was also less likely to be influenced by the presence of a filter when hypopnea events were simulated than apnea events with added UIAL. Yet, APAP algorithm performance was worse during simulated hypopnea events than in apnea events without the use of filters.

At fixed CPAP pressure levels, the measured airway pressure without a filter was within 6% of the set pressure with and without UIAL according to the ISO 80601-2-70:2020 standard. The added filter resistance had a significant impact on delivered end-expiratory pressure. All filters had an effective airway pressure measured more than 6% below the set pressure. The delivered end-expiratory pressure was also severely influenced by the presence or absence of UIAL. In fixed CPAP mode, the actual measured end-expiratory pressure was up to 30% lower than the pressure level set on the device when UIAL were added. This might have a large clinical impact. Depending on how positive airway pressure titration is performed, a drop of the pressure of 1–2 cmH_2_O might already increase the index of residual apnea and therefore alter therapy efficacy. There is however no consensus to define such a clinical relevance threshold. To ensure treatment efficacy, prescribers and healthcare providers must therefore pay utmost attention to UIAL, and mask fit. They must ensure the correction of potential UIAL or consider CPAP prescription adjustment accordingly if UIAL cannot be corrected. Filter choice is also paramount as the pressure drop is almost twice as much for the GVS™ ECO filter™ compared to the GVS™ Medguard™ filter.

The main limitation of our experiments is that we did not test filter resistance before running the scenarios. This could explain why the Virobac II performed the worst, yet not being advertised as the filter with the most airflow resistance from our sample according to the manufacturer's information. Indeed, this filter might be more resistant to airflow than advertised. Manufacturers' data of filter characteristics depend on how they were tested, and specific procedures are not known. In real conditions, filter resistance also varies over time as moisture builds up. For example, Vyaire™ specifies that the AirLife™ filter resistance increases from 0.54 to 0.90 cmH_2_O at 60 L/min after 48 h. This emphasis on the algorithm's ability to detect obstructive respiratory events is likely to be altered over time. The results of this study are therefore only applicable to new and dry filters. Manufacturers also recommend changing the filter once a day, which represents a large number of consumables and might not be financially and logistically realistic in the long term.

The results of this bench study are however limited to the Philips Respironics™ APAP mode which relies on forced oscillation technique to determine event type. Some other manufacturers use different methods, such as short pressure increase, and different algorithms. Only one pulmonary mechanic was also tested in a controlled environment and does not represent real-life conditions. Therefore, the results indicate that APAP performance might be altered using an additional HEPA filter, but further investigations must be performed to assess other devices and scenarios.

The use of an additional HEPA filter should be done according to the recommendation of the local regulation body. If a filter is used, special attention must be paid to avoid any UIAL to prevent potential performance deterioration.

## Conclusion

The APAP algorithm was still able to detect and react to simulated respiratory events with additional HEPA filters when UIAL were avoided. However, the combined effects of added filter resistance and UIAL severely impacted APAP performance and delivered airway pressure. At fixed CPAP level, the additional HEPA filters significantly impacted delivered pressure. Filter choice is also paramount as the impact on pressure drop can be up to two times bigger with the less and the most resistant filter. Further investigations are needed for other devices and real-life scenarios. The use of an additional filter is still debated and must be done according to the recommendation of your local regulation body. If used, utmost attention must be paid on avoiding UIAL.

## Data availability statement

The raw data supporting the conclusions of this article will be made available by the authors, without undue reservation.

## Ethics statement

Ethical review and approval was not required for this study in accordance with the local legislation and institutional requirements.

## Author contributions

Literature search and data collection: NC, LF, and PY. Study design: NC, OC, and J-BM. Analysis of data and manuscript preparation: NC. Review of manuscript: NC, LF, PY, J-BM, JD, and OC. All authors contributed to the article and approved the submitted version.

## Funding

The open access publication fees are covered by the institution of the corresponding author (HESAV).

## Conflict of interest

The authors declare that the research was conducted in the absence of any commercial or financial relationships that could be construed as a potential conflict of interest.

## Publisher's note

All claims expressed in this article are solely those of the authors and do not necessarily represent those of their affiliated organizations, or those of the publisher, the editors and the reviewers. Any product that may be evaluated in this article, or claim that may be made by its manufacturer, is not guaranteed or endorsed by the publisher.
